# A “Turn-On” Fluorescence Copper Biosensor Based on DNA Cleavage-Dependent Graphene Oxide-dsDNA-CdTe Quantum Dots Complex

**DOI:** 10.3390/s18082605

**Published:** 2018-08-09

**Authors:** Liyun Ding, Bing Xu, Tao Li, Jun Huang, Wei Bai

**Affiliations:** 1 National Engineering Laboratory for Fiber Optic Sensing Technology, Wuhan University of Technology, Wuhan 430070, China; xbwhut2018@163.com (B.X.); linht_x@outlook.com (T.L.); hjun@whut.edu.cn (J.H.); 2 School of Information Engineering, Hubei University of Chinese Medicine, Wuhan 430065, China; baiwei027@126.com

**Keywords:** copper ions detection, dsDNA-graphene oxide, CdTe quantum dots, fluorescence recovery

## Abstract

A novel “turn-on” fluorescent copper biosensor is developed successfully based on the graphene oxide (GO)-dsDNA-CdTe quantum dots (QDs) complex via chemical crosslink method. The optical and structure properties of GO-dsDNA-CdTe QDs complex are studied by fluorescence (FL) spectra and transmission electron microscopy (TEM) in detail. It is demonstrated that the fluorescence quenching of CdTe QDs is a process of fluorescence resonance energy transfer (FRET) due to the essential surface and quenching properties of two-dimensional GO. Copper ions induce the catalytic reaction of DNA chain and irreversibly break at the cleavage site, which will cause the G-quadruplex formation, moreover further result in the CdTe QDs separated from GO and restored its fluorescence. Therefore, a significant recovery effect on the fluorescence of the GO-dsDNA-CdTe QDs complex is observed in the presence of copper ions. The fluorescence responses are concentration-dependent and can be well described by a linear equation. Compared with other metal ions, the sensor performs good selectivity for copper ions.

## 1. Introduction

Copper ions (Cu^2+^) are the most abundant essential nutrient in nature except zinc and iron ions, which plays a very important role for biological functions as a constituent of some enzymes [1]. However, excess Cu^2+^ is highly toxic to organism due to disordering gastrointestinal balance or damaging the kidneys and liver [2], as well as listed as priority pollutant in nature. Although several methods are currently employed to detect copper ions based on atomic absorption spectroscopy (AAS) [3], inductively coupled plasma mass spectrometry (ICP-MS) [4], UV-Vis spectroscopy [5], chemiluminescence detection [6] and electrochemical techniques [7,8,9,10], those methods generally need complex preparation process and precision instruments so that their practical applications are tremendously limited. While the use of fluorescent sensors offers distinct advantages, including high sensitivity, good selectivity, time efficiency and the possibility of performing real-time analysis. It is reported that the fluorescent sensors for copper ions detection based on semiconductor quantum dots [11,12], metal nanoclusters [13], upconverting luminescent nanoparticles and organic dyes [14,15,16,17], Ag nanoparticles [18], metal-organic compounds [19] and electrochemical means [20,21]. However, there are still the problems for these fluorescence technologies such as selectivity, water solubility, stability and potential toxicity.

DNAzymes have been regarded as novel and ideal platforms in sensing due to their catalytic ability or binding activity toward specific cofactors, including high binding affinity, easy accessibility and outstanding stability under harsh conditions [22]. So far, numerous sensitive and selective methods based on DNAzyme have been found for Cu^2+^ [23], Pb^2+^ [24,25,26,27,28], Hg^2+^ [29] and K^+^ [30] detection, respectively. The DNAzyme can be complementary to a specific substrate sequence, the substrate chain is cleaved into two fragments when metal ions are present as cofactors [31]. However, traditional DNAzymes-based fluorescent sensors require two or more quenchers labeled at one DNA strand in order to reduce the background, which will inevitably cause complicated or expensive operation and weaken the performance of DNAzymes [32]. It is necessary and urgent to focus on the designing label-free fluorescent sensors based on binding or dynamic cleavage-dependent fluorescence (FL) activation [33].

Recently, two-dimensional graphene-based materials have emerged as promising and novel platforms in sensors due to their unique surface and electronic properties [34]. In particular, graphene can act as an efficient quencher of an organic fluorophore or inorganic luminescent nanomaterial based on either an energy transfer or electron transfer process [35]. As a consequence, graphene has shown great potential in constructing fluorescent DNA biosensors by means of the external competition-dependent graphene/DNA interaction. In this work, by combining catalytic turnover inherent to DNAzymes and subsequent cleavage-induced G-quadruplex formation, a labeled Cu^2+^ fluorescent sensor based on GO-dsDNA-CdTe QDs complex constructed by Cu^2+^ specific DNAzyme was prepared, where CdTe quantum dots as fluorophore were labeled on DNAzyme and graphene oxide as the quencher. The structural transformation of GO-dsDNA-CdTe QDs complex was demonstrated by the change of the fluorescence intensity of CdTe quantum dots. When the CdTe quantum dots and graphene oxide were cross-linked with the end of functional DNAzyme, the CdTe quantum dots show a lower fluorescent in aqueous media. However, the addition of Cu^2+^ makes the fluorescence of the CdTe quantum dots change from “turn off” to “turn-on.” Meanwhile, the selective of the GO-dsDNA-CdTe QDs complex for Cu^2+^ were studied. Compared with other methods, this method has the characteristics of simple operation, high sensitivity and good selectivity.

## 2. Experimental

### 2.1. Materials and Methods

NaCl, KCl, CdCl_2_, Pb(NO_3_)_2_, MgCl_2_, Zn(NO_3_)_2_, CaCl_2_, FeCl_3_, CoCl_2_, Na_2_HPO_4_ and NaH_2_PO_4_ were of analytical grade and bought from Sinopharm Chemical Reagent Co, Ltd. (Shanghai, China). Cu^2+^ substrate sequence (Cu-Sub) (5′-AGCTTCTTTCT-AATACGGCTTACC-3′) (ssDNA) and enzyme sequence (Cu-Enz) (5′-GGTAAGCCTGGGCCTCTTTCTTTTTAAGAAAG-AAC-3′) (cDNA) were bought from Wuhan TianyiHuiyuan Life Science & technology Inc., Ltd. (Wuhan, China). Different pH values of phosphate buffer solutions (PBS, 0.05 M) were prepared using the stock solution of Na_2_HPO_4_ and NaH_2_PO_4_ with various volume ratios. GO was prepared in accordance with the well-documented Hummers methods [36] (1958, hummers). The water-soluble CdTe QDs are prepared by One-pot synthesis [37].

### 2.2. Apparatus

The F-4500 fluorescence spectrophotometer (Hitachi, CO., Tokyo, Japan) was used to measure the fluorescence of GO-dsDNA-CdTe QDs complex at excitation wavelength of 473 nm. UV-vis spectra were collected by the UV-2450 spectrophotometer (Shimad-zu, CO., Kyoto-fu, Japan). The morphology was studied in detail by a JEM-2100F STEM/EDS TEM (JEOL, Tokyo, Japan).

### 2.3. GO-dsDNA-CdTe QDs Complex Synthesis

25 mM EDC and 100 mM NHS was added to the GO solution in the PBS solution (10 mM Na_2_HPO_4_/NaH_2_PO_4_, 0.3 m NaCl and pH = 7.4) to activate the carboxyl group on GO sheet and dissolved and kept for 2 h. Then, 4 uM ssDNA was added to the activated carboxyl groups of GO solution (1 mL of 1 g/L) and cultivated overnight. Subsequently 4 uM cDNA was added to the GO-ssDNA solution and kept for 1 h at 95 °C in a water bath and then natural cooling to room temperature. 10 mM PBS solution was used to centrifuge and wash cDNA, ssDNA and GO solutions, respectively and then obtained GO-dsDNA product was stored at 4 °C for the future use. 25 mM EDC and 100 mM NHS was added to the CdTe QDs solution and cultivated for 2 h. GO-dsDNA was added the above CdTe QDs solution and incubated for 24 h, finally GO-dsDNA-CdTe complex was obtained.

## 3. Results and Discussion

### 3.1. Principle of the Cu^2+^ Sensing System

**Figure 1:**
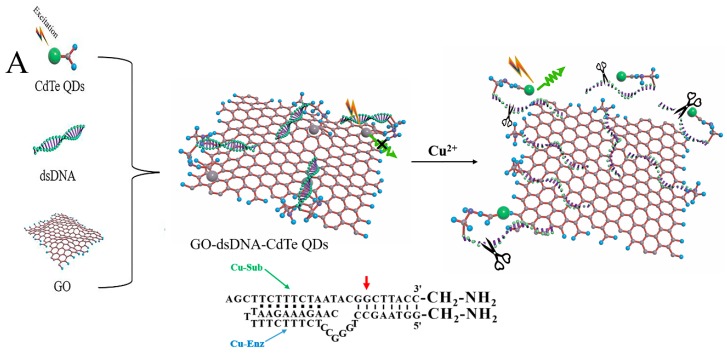

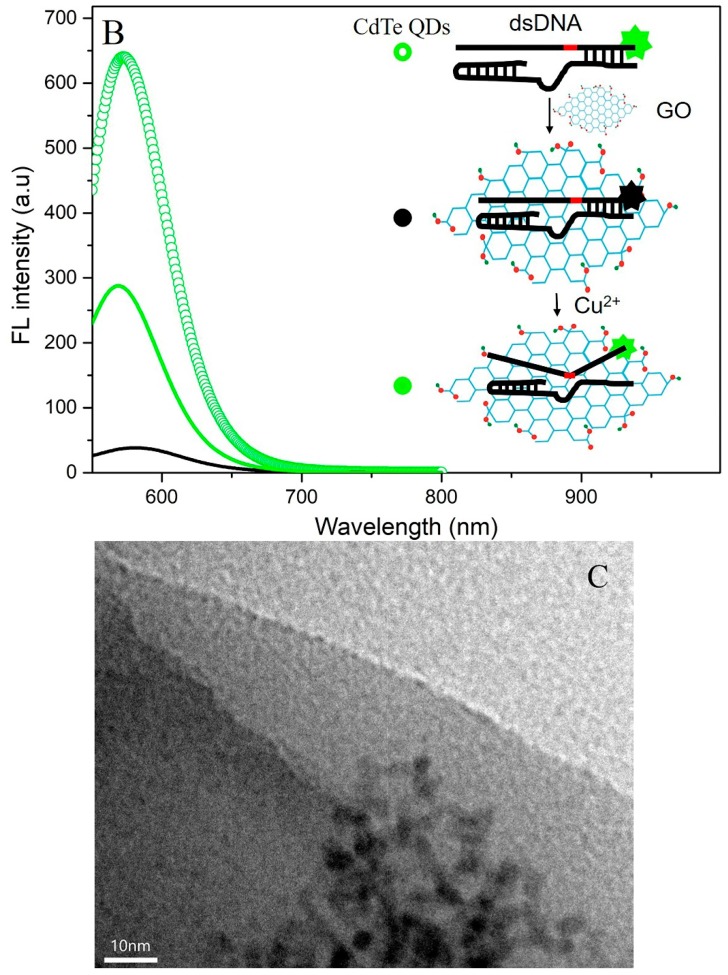
(**A**) Illustration of the operating principle of the “turn-on” fluorescent sensor for Cu^2+^ detection; (**B**) The FL spectra of GO-dsDNA-CdTe QDs complex in the absence or presence of Cu^2+^; (**C**) TEM images of GO-CdTe nanocomposite.

The design of the Cu^2+^ biosensor based on GO-dsDNA-CdTe complex is shown in Figure 1A. The system is a combination of two components that formed a self-assembled complex. The first component was the Cu^2+^ dependent DNAzyme containing a 3′-FAM-modified Cu-Sub and a Cu-Enz, which served as an effective recognition site. The second component was the FL transduction unit of graphene, which was employed as an independent quencher to suppress background signal. As shown in Figure 1B, initially, the CdTe QDs is fixed to one end of the ssDNA chain and CdTe QDs exhibits the strong fluorescence emission at 540 nm without GO. And as the GO mixed with the CdTe QDs–dsDNA, the fluorescence emission of the CdTe QDs will be quenching. It is because that sp2 bonded carbon atoms of GO and aromatic nucleobases of dsDNA will reveal π-π stacking interactions, which makes the distance between GO and CdTe QDs smaller than 10 nm [37,38,39,40] and such a short distance helps transfer the energy from excited state of CdTe QDs to GO. Therefore, the fluorescence emission of the CdTe QDs will be quenched. In the presence of Cu^2+^, the DNA substrate was irreversibly cleaved as a result of the specific DNAzyme-Cu^2+^ interaction, disturbing the self-assembled graphene–DNAzyme conformation. As a result, the quantum dots separated from the GO and restored the fluorescence. Hence, the GO-dsDNA-CdTe QDs complex could act as the sensor platform for Cu^2+^ detection. The FL spectra of GO-dsDNA-CdTe QDs complex in the absence or presence of Cu^2+^ is shown in Figure 1B. It can be demonstrated that the FL intensity of GO-dsDNA-CdTe QDs complex get effectively enhance when the Cu^2+^ was mixed with GO-dsDNA-CdTe QDs complex. It is verified that the CdTe nanoparticles are well dispersed and immobilized on GO surface as shown in the Figure 1C.

### 3.2. Optimization of Experimental Conditions

**Figure 2:**
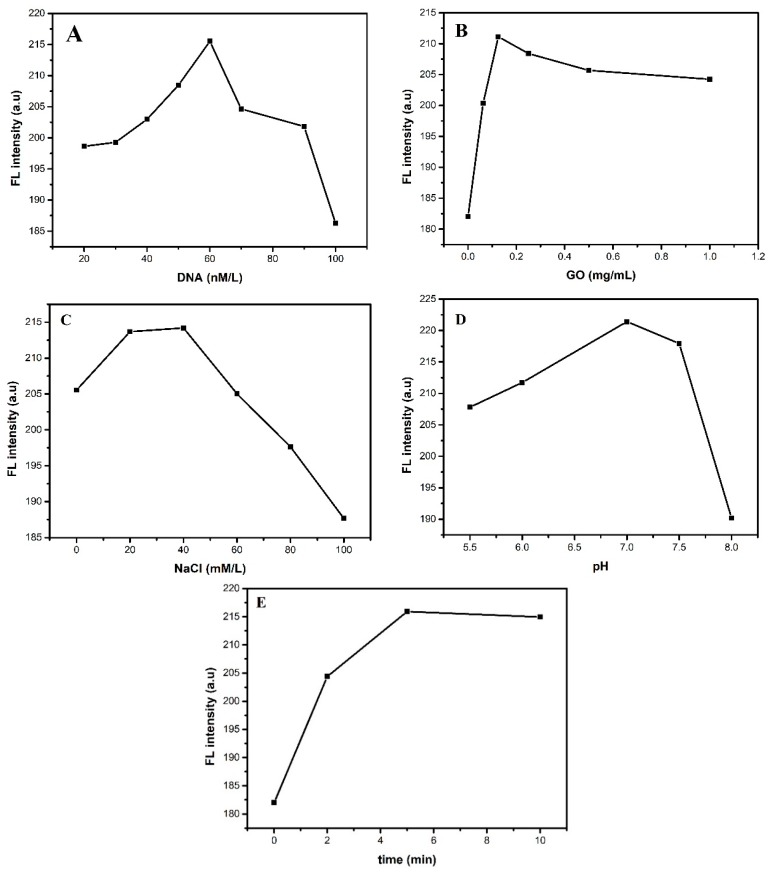
The FL intensity of (**A**) in PBS buffer (50 mmol/L, pH 7.0) containing 40 mmol/L NaCl, 0.125 mg/mL GO, different concentrations of DNA for 5 min; (**B**) in PBS buffer (50 mmol/L, pH 7.0), containing of 40 mmol/L NaCl, different concentration of GO and 60 nmol/L DNA for 5 min; (**C**) in PBS buffer (50 mmol/L, pH 7.0) containing different concentration of NaCl, 60 nmol/L DNA, 0.125 mg/mL GO; (**D**) in PBS solutions (50 mol/L) with different pH values containing 40 mmol/L NaCl, 60 nmol/L DNA, 0.125 mg/mL GO for 5 min; (**E**) in PBS solution (50 mmol/L, pH 7.0) containing 40 mmol/L NaCl, 60 nmol/L DNA and 0.125 mg/mL GO for various time.

In order to improve the performance of the sensor for the Cu^2+^ detecting, the usage conditions of GO-dsDNA-CdTe QDs complex must be optimized. The concentration of DNA is an important factor affecting the performance of fluorescent sensor. Figure 2A shows the relationship between the DNA concentration and the FL intensity. It can be seen from Figure 2A that the FL intensity of the system increases continuously with the DNA concentration increasing from 0 to 60 nmol/L with the concentration of Cu^2+^ is 5 umol/L, while FL intensity decreased when DNA concentration increases from 60 to 100 nmol/L. When the concentration of DNA is low, increasing the amount of DNA is equivalent to increase of CdTe QDs connected with DNA, so the FL intensity of CdTe QDs enhanced obviously. However, the CdTe QDs released from the cleaved DNA and meet the GO sheet again if the concentration of DNA is excessive; resulting in the fluorescence was quenched. Therefore, the concentration of DNA in this experiment is chosen as 60 nmol/L.

The concentrations of GO as the quencher of the fluorescence probe affects the probe’s performance. Figure 2B shows that the fluorescence signal increased with increasing concentration of GO and reached the maximum intensity at 0.125 mg/mL with the concentration of Cu^2+^ is 5 umol/L. The reason is that the free CdTe QDs from the cleaved DNAzyme could meet GO again when the concentration of GO was too much. Thus, the optimum concentration of GO in this experiment is 0.125 mg/mL.

The ionic strength is a measure of the concentration of each ion in the solution. The catalytic activity of the DNA enzyme and the stability of the hybridization will be affected by the excessively ionic strength. In this study, ionic strength is regulated by the concentration of NaCl. In order to ensure the stability of the Cu^2+^ ion-specific DNA enzyme during the experiment, the high concentration of ion intensity in the buffer solution is very necessary. It can be seen from Figure 2C that the FL intensity increases continuously with the DNA concentration increasing from 0 to 40 mmol/L, while FL intensity decreased as DNA concentration increases from 40 to 100 mmol/L with the concentration of Cu^2+^ is 5 umol/L. Thus, the optimum concentration of NaCl is chosen as 40 mmol/L.

The pH value is one of the most important parameters influencing the capability of the sensor. On the one hand, strong acid condition will reduce the solubility of DNA, due to damaging the structure of DNA and bringing about the inactivation of DNA, which will eventually affect the number of DNA hybridized with CdTe QDs and further affect the fluorescence performance of CdTe QDs. On the other hand, Cu(OH)_2_ precipitation of Cu^2+^ ions will be formed under strong alkaline environment. Therefore, the range of pH is set from 5.5 to 8.0 in this experiment. As shown in Figure 2D, the FL intensity of the fluorescence signal increased gradually with pH and reached the maximum at pH = 7.0 and then decreased when pH further increased to 8.0. Thus, the optimum value of pH for Cu^2+^ detection is chosen as 7.

The reaction time of DNA catalyzed by Cu^2+^ ion depends on the concentration of Cu^2+^ ion. The Cu^2+^ ion concentration (100 μmol/L) is chosen as reference to analyze the reaction. The effect of test time on the performance of GO-dsDNA-CdTe QDs complex fluorescent probe is shown in Figure 2E. With the increase of reaction time from 0 to 10 min, the FL intensity increases rapidly and reaches the maximum at 5 min, then remained the same after 5 min. Therefore, 5 min is selected as the best time to detect the concentration of Cu^2+^ ions.

### 3.3. The Analytical Performance of the Sensor

**Figure 3:**
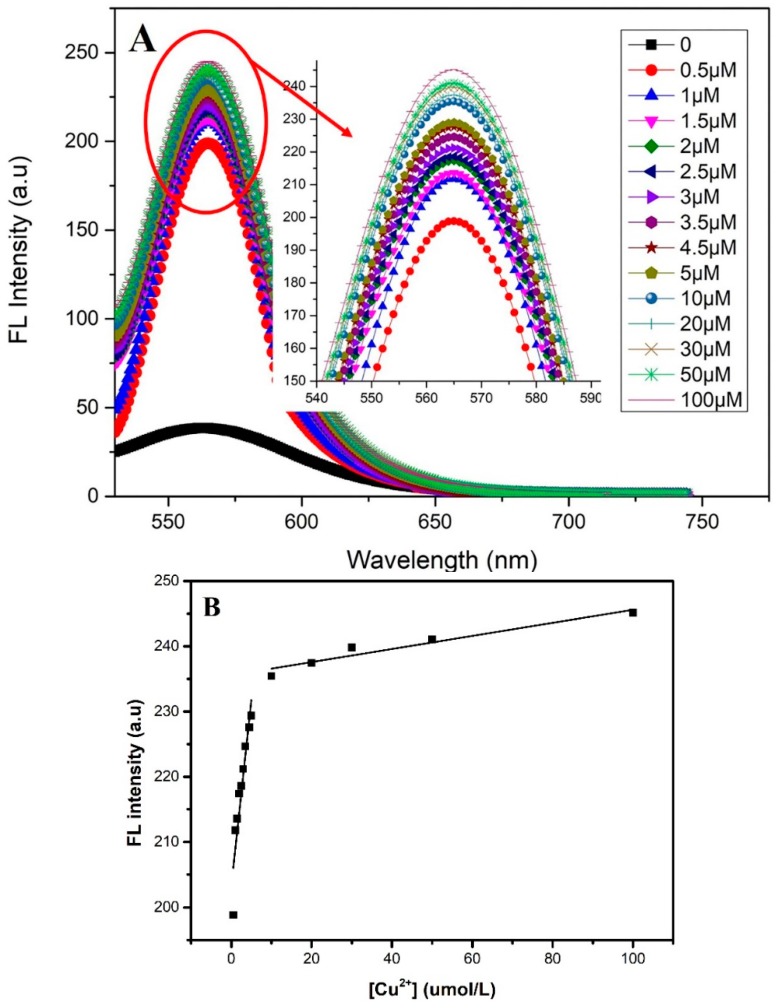
(**A**)The fluorescence spectra of the GO-dsDNA-QD ensemble assay upon addition of various concentration of Cu^2+^. Inset: Local fluorescence amplification; (**B**) The relationship between the fluorescence intensity and Cu^2+^ concentration.

Analytical performance is a key factor to measure a practical sensor. There are the fluorescence spectra of the GO-dsDNA-CdTe QDs complex upon addition of various concentration of Cu^2+^ show in the Figure 3A. With the addition of Cu^2+^, the FL intensity significantly increased, suggesting that the DNAzyme was activated and catalyze the cleavage of the hairpin substrate strand into inter-molecularly hybridized double-strand DNA with much less stability, resulting in the CdTe QDs far away from GO, thus producing an increased fluorescence signal. The FL intensity increased with the increase of Cu^2+^ concentration. It reached a plateau when the Cu^2+^ concentration increased to 5 μmol/L. It is mainly because the number of GO-dsDNA-CdTe QDs complex is limited, adding a small amount of Cu^2+^ ion can make it full contact with the GO-dsDNA-CdTe QDs complex and release CdTe QDs quickly, so that the fluorescence intensity fast recovered. Excessive Cu^2+^ may decrease the contact with the GO-dsDNA-CdTe QDs complex and decrease the speed of CdTe QDs released, so the FL intensity rises slowly. Figure 3B presented the calibration curve of the sensor in the range of 0.5 to 100 μmol/L. In the linear response range (0.5–5 μmol/L, 5–100 μmol/L) and the calibration curve can be expressed by the equation $I=5.724{\mathrm{Cu}}^{2+}+203.15755$(*R*^2^ = 0.865) and $I=0.10034{\mathrm{Cu}}^{2+}+235.574$ (*R*^2^ = 0.919). The sensor shows a great linear relationship between the FL intensity and the concentration of Cu^2+^ because of the super quenching capability of GO and the significant difference in the affinity of dsDNA toward Cu^2+^ and GO.

**Figure 4:**
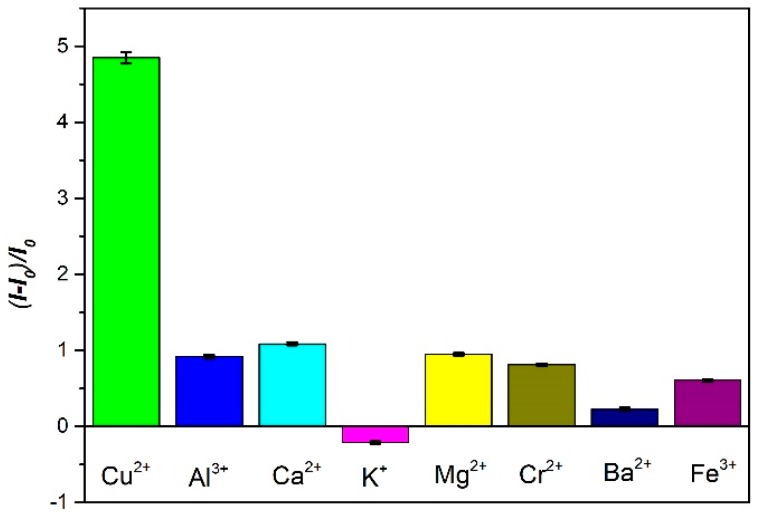
The selectivity of Cu^2+^ analysis using the GO-dsDNA-CdTe QD’s sensor.

In order to investigate the effective selectivity of GO-dsDNA-CdTe QDs complex to Cu^2+^ ions, the environmentally relevant metal ions solution of the same concentration (100 μmol/L), including Ag^+^, Ca^2+^, Cd^2+^, Pb^2+^, Fe^3+^ and K^+^, was respectively prepared under the best detection conditions. Here, we define a signal-to-background ratio (SBR) as $\left(I-I_{0}\right)/I_{0}$ to eliminate the influence of the original background intensity, which was used to estimate the FL response, where *I* and *I*_0_ were the FL signals of the CdTe QDs in the presence and absence of Cu^2+^, respectively. Selectivity is defined as SBR of the probe for Cu^2+^ detection against other metal ions. As shown in Figure 4, although any heavy metal ions except for Cu^2+^ are added in the solution, the FL intensity of GO-dsDNA-CdTe QDs complex still did not change. This result showed that compared with other metal ions, the GO-dsDNA-CdTe QDs complex has great selectivity to Cu^2+^ because of the G-quadruplex structure between dsDNA and Cu^2+^.

## 4. Conclusions

In this paper, a fluorescent Cu^2+^ sensor based on GO-dsDNA-CdTe QDs complex were prepared by chemical crosslinking method. We demonstrated the fluorescence emission GO-dsDNA-CdTe QDs complex can be “turned-on” by the presence of Cu^2+^ based on the FRET mechanism between CdTe QDs and GO. The optimum experiments conditions were determined, including the concentration of DNA (60 nmol/L), GO (0.125 mg/mL), NaCl (40 mmol/L) and pH (7) of solution and the best detection time is 5min. A linear correlation between Cu^2+^ and FL intensity was obtained with $I=5.72{\mathrm{Cu}}^{2+}+203.16$ (*R*^2^ = 0.865) in the range from 0.5 to 5 μmol/L. The detection limit of the sensor presented is 0.5 uM (*S*/*N* = 3). The results show that the GO-dsDNA-CdTe QDs complex has good recognition ability of Cu^2+^. The sensing mechanism is suitable for the detection of other heavy metal ions, small molecules and biomolecules.
